# An Insect-Inspired Terrains-Adaptive Soft Millirobot with Multimodal Locomotion and Transportation Capability

**DOI:** 10.3390/mi13101578

**Published:** 2022-09-22

**Authors:** Han Huang, Yu Feng, Xiong Yang, Liu Yang, Yajing Shen

**Affiliations:** 1 Department of Biomedical Engineering, City University of Hong Kong, Hong Kong 999017, China; 2 Shenzhen Research Institute of City University of Hong Kong, Shenzhen 518057, China; 3 Department of Electronic and Computer Engineering, The Hong Kong University of Science and Technology, Hong Kong 999077, China

**Keywords:** soft robot, magnetic driven, multimodal locomotion, cargo delivery

## Abstract

Inspired by the efficient locomotion of insects in nature, researchers have been developing a diverse range of soft robots with simulated locomotion. These robots can perform various tasks, such as carrying medicines and collecting information, according to their movements. Compared to traditional rigid robots, flexible robots are more adaptable and terrain-immune and can even interact safely with people. Despite the development of biomimetic principles for soft robots, how their shapes, morphology, and actuation systems respond to the surrounding environments and stimuli still need to be improved. Here, we demonstrate an insect-scale soft robot with multi-locomotion modes made by Ecoflex and magnetic particles, which can be actuated by a magnetic field. Our robot can realize four distinct gaits: horizontal tumbling for distance, vertical tumbling for height, imitation of gastropod writhing, and inchworm-inspired crawling for cargo delivery. The soft compliant structure and four locomotion modes make the robot ideal for maneuvering in congested or complex spaces. In addition to linear motion (~20 mm/s) and turning (50°/s) on a flat terrain, the robot can also maneuver on various surface conditions (such as gaps, smooth slopes, sand, muddy terrain, and water). These merits, together with the robot’s high load-carrying capacity (5 times its weight), low cost, obstacle-crossing capability (as high as ~50% its length), and pressure resistance (70 kg), allow for a wide variety of applications.

## 1. Introduction

Soft robots gain advantages in terms of adaptability to complex environments [1], precise manipulation of small objects [2,3], movement in confined spaces [4], and high degrees of freedom of movement due to their deformable qualities and the diversity of drive methods [5,6,7,8]. These advantages have led to an increasing interest in their potential application in biomedicine, bioengineering, bionics, automation, and industry. With the development of soft composites and various contact/non-contact drive technologies, the movement patterns and functional realization of soft robots have been increasingly extended [9]. Soft robots can be deformed and moved by expanding, contracting, bending, twisting, or combining in response to different actuation inputs [10]. So far, soft robots have also been driven by increasingly diverse methods, such as pneumatic pressure [11], light [12], magnetic fields [13], and electricity [7,14]. Compared to other actuation methods, the magnetic drive has the following advantages [15,16]: (1) non-contact—due to the inherent penetration of the magnetic field, magnetically driven robots can operate in a variety of confined spaces without external airways or interventional drive devices, extending the application scenarios; (2) biosafety—the drive field is small (typically less than 1 T) and penetrates well, making it harmless to humans; and (3) the development of magnetic control technology—the current method of generating the required magnetic field through current-controlled electromagnetic coils or motion-controlled permanent magnets is well established, ensuring a flexible drive strategy for soft robots. Various forms of magnetically driven soft robots have emerged, such as rolling [17], creeping [18], and multilegged robots [13]. However, these robots have certain drawbacks that affect their use in complex environments, such as high overall or local stiffness and limited deformation capabilities [19]; limitations in the actuation form, resulting in poor robustness and loss of mobility when overturned or folded [13]; high demands on the interface of movement and loss of mobility when encountering obstacles or slick interfaces [15]; and poor load-carrying capabilities—some do not have the capacity to carry or can only maintain a limited load amount [20].

The locomotion mechanisms of insects with strong environmental adaptability have inspired the design of robots. A desert spider can tumble by curling its body to obtain a movement rate of more than three times the usual crawl [21]; the locomotion of the inchworms gives it a high degree of inclusivity in terms of directionality of movement (360°) and slope of the interface (45°) [22]; and snails can wriggle across rugged and complex interfaces [18]. Mimicking the locomotion patterns of these insects can aid the design of flexible robots with environmental adaptability. Kim et al. [23] designed a miniature piezoelectric, ceramic-based, multi-legged robot that imitated the crawling of legged insects by generating high-frequency vibrations through an applied piezoelectric vibrator, enabling the robot to move to generate high-frequency vibrations. Zhang et al. [24] implemented a magnetic multi-legged robot that could crawl forward in a paddling fashion, a motion that mimicked the movement of tsetse. Gu et al. et al. [25] magnetized the robot by rolling it into a cylinder, thus achieving a millipedal-like wave-like gait while switching between crawling and rolling movement modes, depending on the applied magnetic field. Yang et al. [26] designed a soft-bodied millipede robot inspired by a starfish that was able to perform a full range of motion in complex environments. Although these studies mimicking the movement of insects obtained excellent kinematic performance, they place high demands on the rate of change of external drives (PZT actuators, magnetic fields, etc.). Moreover, their crawling distance per second and their height over obstacles do not reach the level of body length. In addition, the materials used to prepare these flexible robots do not allow them to maintain their locomotion under heavy stress (>50 kg).

Although mimicking the movements of insects can bring about improvements in locomotion, each movement has its limitations, such as the difficulty of controlling direction when moving over rugged and uneven surfaces while crawling for stability and terrain adaptation. Based on these issues, robots with multiple locomotor modes can combine the advantages of different locomotor modalities by using them in different situations to achieve optimized locomotor performance.

In this work, we present a bionic insect soft robot with multiple locomotor modes and high adaptability by using conductive magnetic powder and Ecoflex. Through designing the magnetization direction and shape of the robot, the following functions were achieved: (1) mimicking the locomotor modes of a spider, the 12 mm-long soft robot (0.3 g) achieved a relative movement speed of ~0.9 BL (body length)/s, driven by a low-frequency magnetic field (1 Hz), and could overturn obstacles up to 50% of its body length; (2) mimicking the creeping movement of an inchworm and the crawling of a snail, there was (3) no effect on locomotion after stepping on the robot with the whole body weight of an adult man (~70 kg, approximately 230,000 times heavier than the robot); and (4) loads of up to 5 times the robot’s body weight for locomotion.

## 2. Materials and Methods

### 2.1. Fabrication of the Soft Millirobot

The Ecoflex stock solution was prepared by mixing 7 g of Ecoflex-A with 7 g of Ecoflex-B for 5 min. Then, 6 g of iron oxide powder was added and stirred thoroughly for five minutes. This was followed by evacuation in a vacuum pump for 10 min. It was then poured into a 3D printed mold and cured in a parallel magnetic field formed between two equal-sized permanent magnets for 40 min. As shown in Figure S1, due to the viscosity of the Ecoflex, the mixture remains evenly during the curing. Once the curing was complete, the mold was removed and cut into 12 mm × 5 mm sheets. The previously prepared mixture was reintroduced into the mold, and the cutout sheet was immersed so that it formed approximately 20° to the horizontal plane. It was cured in the same parallel magnetic field for 40 min, demolded, and the excess trimmed off. The internal magnetization direction distribution of the final formed structure is shown in Figure S2. The SEM images of the robot’s cross-sectional view is shown in Figure S3.

### 2.2. Magnetization Characterization of the Soft Millirobot

**Figure 1:**
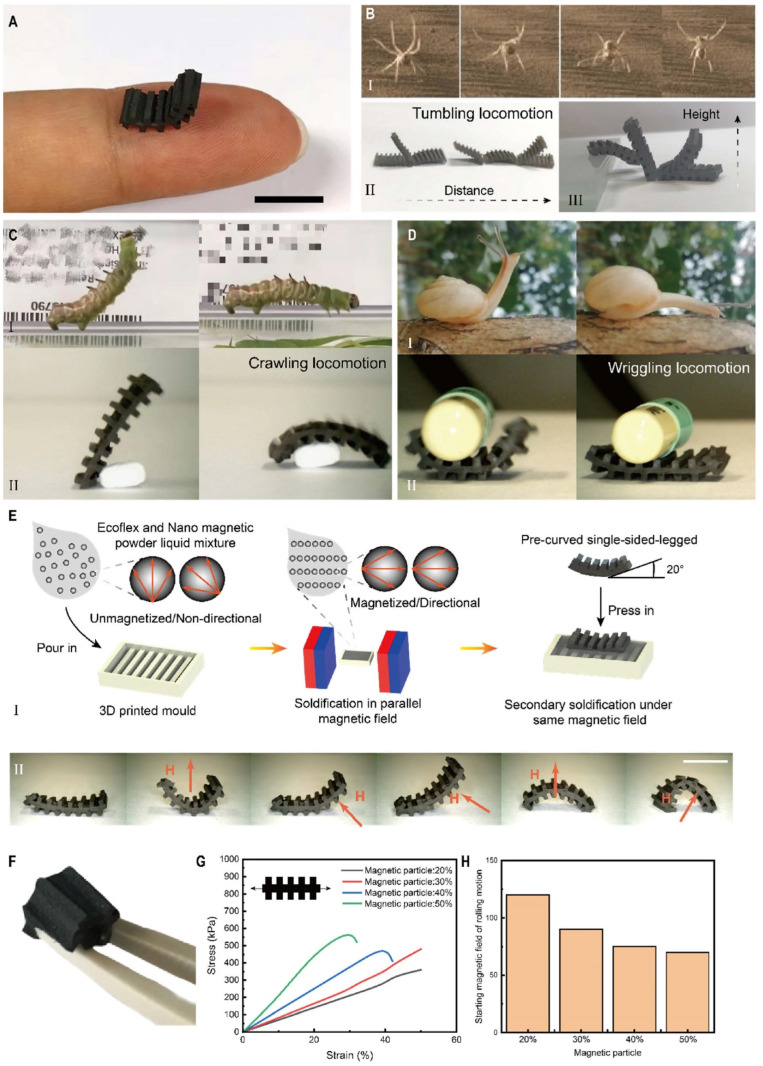
Insect-inspired soft robot with multimodal locomotion modes. (**A**) The robot moves through a finger (scale bar: 10 mm). (**B**) A tumbling locomotion that mimics the movement of a spider, allowing for displacement and overturning heights: (I) the rolling motion of spiders, by curling their knuckles to form a structure with multiple contact points, obtains a rapid locomotion far beyond that of crawling; (II) tumbling locomotion for distance movement; (III) tumbling locomotion for height movement over obstacles. (**C**) (I) The crawling locomotion movement of an inchworm, which has good steering performance; (II) a crawling locomotion that mimics the movement of an inchworm, allowing it to remain stable and grasp the drug. (**D**) (I) The wriggling locomotion movement of a snail, which is highly adaptable to the terrain; (II) a wriggling locomotion that mimics the movement of a snail. (**E**) The manufacturing process of soft robots: (I) A 1:1 mixture of Ecoflex-1 and Ecoflex-2 solutions was mixed with iron oxide powder in a 7:3 mass ratio. After thorough mixing, it was added to a mold made by 3D printing and cured in a parallel magnetic field. After the first curing was completed, the flexible layer with a leg-like structure on one side was demolded and cut into thin rectangular layers of ~12 mm × ~5 mm. The cured layer was re-immersed in a mold filled with a liquid Ecoflex-iron powder mixture, kept at 20° from horizontal at both ends, and cured under the same magnetic field, resulting in a soft robot with a bilateral leg-like structure; (II) due to the directional magnetic chains formed within the soft robot, the robot undergoes deformation to align the whole with the magnetic flux under the influence of an applied magnetic field. Different responses were achieved when the direction and strength of the magnetic field were different. H represents the internal pulling force. (Scale bar: 2 cm) (**F**) Fully flexible soft robot that can be deformed at any angle. (**G**) Strain–stress curves for the flexible robots with different formulations. (**H**) Minimum rotational start-up magnetic field strength for soft robots with different magnetic particle mass fractions. The horizontal coordinate is the mass fraction of magnetic powder in it.

The magnetization (magnetic moment density) of the robot with different magnetic field strengths was measured by a vibrating sample magnetometer (DMS 1660, ADE Technologies). As shown in Figure 1H, the magnetization of the iron oxide increased from 0 to 70 emu/g as the applied magnetic field changed from 0 to 20,000 Oe.

### 2.3. Magnetic Control Methods for the Soft Millirobot

The control of the flexible robot uses mainly permanent magnets, the magnetic field distribution of which is shown in Figure S4. By rotating the permanent magnets, a rotating magnetic field can be obtained to control the soft robot in a tumbling motion. By simultaneously moving in the *x* and *z* axes, the soft robot can be controlled to mimic the crawling of an inchworm and a snail.

### 2.4. Gripping Capability of the Millirobot

The robot’s grasping function was achieved by mimicking the movement of an inchworm. Using square permanent magnets for control, the alternating proximity of the front and rear feet increases the friction of the robot’s feet against the ground through the attraction of the magnets and thus the creeping movement through deformation. The ends of the robot did not leave the ground during the entire writhing process and continued to wrap around the delivered tablets.

## 3. Results

### 3.1. Design and Manufacturing of the Soft Millirobot

Insects have always been an important source of inspiration for robot design due to their simple, flexible bodies and highly adaptable locomotion. In order to improve the actuation performance and simplify the manufacturing of the robot, we have designed a structurally simple soft robot to achieve a variety of insect-like movements. A prototype robot of 12 mm by 5 mm, consisting of a slightly upwardly curved body (~1 mm thick) and leg-like structures (~1.5 mm high) symmetrically positioned on either side of the body, is shown in Figure 1A, with size comparison using a human finger.

The robot’s shape is mainly determined by a combination of both the movement capacity and the material’s properties. As a microrobot for gastrointestinal applications, we would like the robot to be as small as possible, thus improving its ability to traverse tight environments. However, as mentioned above, the traversing ability of the robot is directly related to its body length (~50% of its body length). Since the height of a healthy gastrointestinal mucosal fold is approximately 5 mm, we chose a body length of 12 mm ×5 mm in order to obtain the best possible traversing ability. After determining the dimensions, we further designed the structure of the robot. Compared to a flaky robot without a three-dimensional structure, the leg structure on both sides allows the robot to be bent more efficiently, reducing the contact area with the ground, reducing the friction of movement, increasing freedom of movement, and adapting better to wet environments (Figure S5). For the ratio between the robot’s leg length and leg spacing, a ratio of 1:1 is a relatively good solution for the flexible legs in terms of movement efficiency and support of the body, according to the available literature. Finally, there is the curvature of the robot, a structure that forms a grip and improves stability when creeping with cargo on its back. The curvature is determined by a combination of the viscosity of the Ecoflex material in its liquid state and Young’s modulus after curing. Due to the inherent elasticity of Ecoflex after curing, angles that are too small cannot be maintained after molding and will form a sheet robot without curvature. During preparation, the viscous liquid Ecoflex adheres to the first cured flakes, limiting the curvature that can be formed. A combination of performance and manufacturing process led us to choose 20° as the curvature of the robot. Ecoflex is a water-, acid-, and alkali-resistant, non-corrosive, physiologically inert polymer. When encapsulated with iron powder, which is a magnetic material, it protects it from the biochemical environment of the intestine. At the same time, the high flexibility of the robot ensures that it is safe for use in the human body and does not cause damage to human tissue.

As shown in Figure 1B–D, by applying a magnetic field, our robot acquires different gaits (tumbling, crawling, and wriggling). This locomotion mechanism uses a single structure to achieve multiple forms of locomotion to address the challenges of multi-terrain mobility and scalability for small robots (Video S1). This different response to different magnetic fields is mainly due to the design of the magnetization distribution during the manufacturing process.

As shown in Figure 1E, a 1:1 mixture of the Ecoflex-1 and Ecoflex-2 solutions was mixed with iron oxide powder in a 7:3 mass ratio. After thorough mixing, it was added to a mold made by 3D printing and cured in a parallel magnetic field. Under the influence of an applied directional magnetic field (~150 mT), the randomly distributed iron oxide powder in Ecoflex was arranged into orientated chains, where the formed magnetic chains tend to coincide with the magnetic field lines. After the first curing was completed, the flexible layer with a leg-like structure on one side was demolded and cut into thin rectangular layers of ~12 mm ×~5 mm. The cured layer was re-immersed in a mold filled with a liquid Ecoflex–iron powder mixture, kept at 20° from horizontal at both ends, and cured under the same magnetic field, resulting in a soft robot with a bilateral leg-like structure. Due to the directional magnetic chains formed within the soft robot, the robot undergoes deformation to align the whole with the magnetic flux under the influence of an applied magnetic field. From there, various kinematic forms are derived, as will be described in detail later on.

The flexibility of the robot is given by the Ecoflex, which is used as an encapsulation carrier. The soft rubber ensures rapid deformation and recovery of the robot due to its excellent mechanical properties. As shown in Figure 1F, the robot has good deformation capabilities. In addition, to investigate the robot’s ability to resist deformation, we measured the stress–strain curve of the robot at different ratios. As shown in Figure 1G, the maximum elongation decreases from ~50% to ~25% and tearing occurs as the mass fraction of the inelastic magnetic particles in the robot material increases from 20% to 50%. At the same time, the start-up magnetic field (tumble) decreases from 120 mT to 70 mT, as shown in Figure 1H. Taking into account the tensile performance and the drive performance, a ratio of 30% was chosen. Robots made of flexible materials not only improve the robustness of the robot but also allow for a variety of movement patterns and the ability to traverse tight areas through the robot’s own deformation.

### 3.2. Tumbling Locomotion for Distance

**Figure 2:**
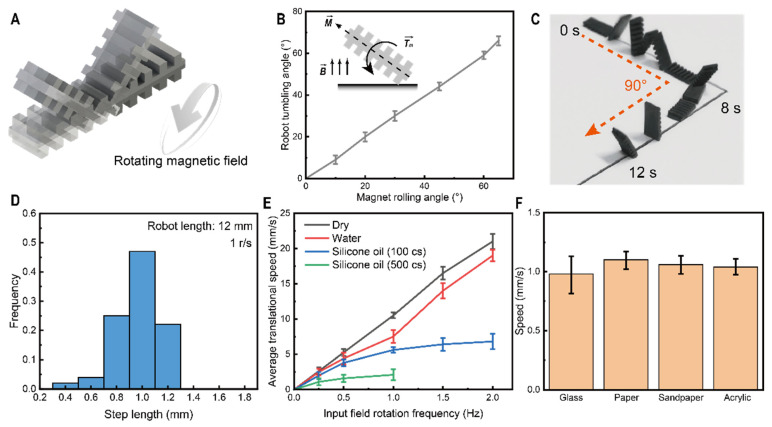
Soft robot with a tumbling-based linear motion. (**A**) Soft robot tumbling motion driven by rotating magnetic fields. (**B**) Relationship between the magnetic field angle and robot rotation angle. (**C**) Fast movement and turning capability of the soft robot. (**D**) Frequency diagram of the soft robot step distribution in a rotating magnetic field at 1 r/s. (**E**) Relationship between the speed of the soft robot movement and the magnetic field speed on substrate surfaces coated with different materials. (**F**) Average rate of movement of soft robots on different substrate surfaces.

Due to the magnetic field applied during the curing process, when the magnetic alignment of the applied field does not coincide with this alignment direction of the robot, a magnetic torque is induced onto the robot, which is realigned with the magnetic field direction by rotation. Applying a continuously rotating magnetic field along an axis of rotation parallel to the horizontal causes the robot to rotate about the same axis, as shown in Figure 2A. In addition, moving the magnetic field along the *y* axis will force the robot to remain aligned with the magnetic field direction through rotation, and result in a continuous forward tumbling motion. To verify the nature of this alignment with the external drive field, we measured the angle of rotation of the applied magnetic field with the angle of rotation of the robot on one end of the axis, as shown in Figure 2B. The two angles were strictly aligned, verifying the feasibility of such a rotating magnetic field to control the robot.

As can be seen from the tumbling process, each magnetic field rotation cycle in the magnetic field drive cycle produces a forward step of slightly less than one time the soft robot’s body length. However, the crawling process inevitably receives a series of perturbations, such as sliding of the support points, dragging of the driving magnetic field, and bouncing as the soft robot resumes its deformation after deformation. These random processes involved in the tumbling dynamics result in a measured step length histogram with a normal distribution, as shown in Figure 2D.

Measuring the average velocity of the robot under various conditions can help to understand the kinematic capabilities of a soft robot driven by a rotating magnetic field. As shown in Figure 2E, since the robot’s tumbling motion strictly adheres to the rotational period of the magnetic field, the speed of movement in dry environments and on surfaces with aqueous substrates increases from 0.25 Hz to 2 Hz and from ~3 mm/s to ~22 mm/s, in proportion to the rotational frequency of the magnetic field. Still, in more demanding motion environments (coated with 100 cs and 500 cs viscous silicone oil-coated substrate surfaces), the higher viscosity and greater resistance cause a phase lag between the robot orientation and the external magnetic field. As the frequency of rotation of the external field increases, the increased resistance causes the robot to oscillate in place rather than tumble forward. This behavior occurs in rotating magnetic fields above 1 Hz, and as the viscosity of the silicone oil used increases, magnetic fields above 1 Hz are unable to drive the robot into motion. Although the different fluids on the substrate surface can affect the robot’s kinematic performance, it has good performance with different substrates. As shown in Figure 2F, there is essentially no difference in the robot’s motion on glass, paper, sandpaper, and acrylic surfaces, all remaining at around 1 mm/s, showing good environmental adaptation.

In addition to straight-line motion in a single direction, turning performance is also an essential part of measuring the robot’s kinematic capabilities. When the robot is in an upright position, and the applied magnetic field is turned at an angle along the *z* axis, the robot will turn using the support point as a fulcrum in order to remain stationary as the magnetic torque has not yet reached equilibrium, thus achieving a turning effect. The process of the soft robot performing the turning motion is shown in Figure 2C.

### 3.3. Tumbling Locomotion for Height

**Figure 3:**
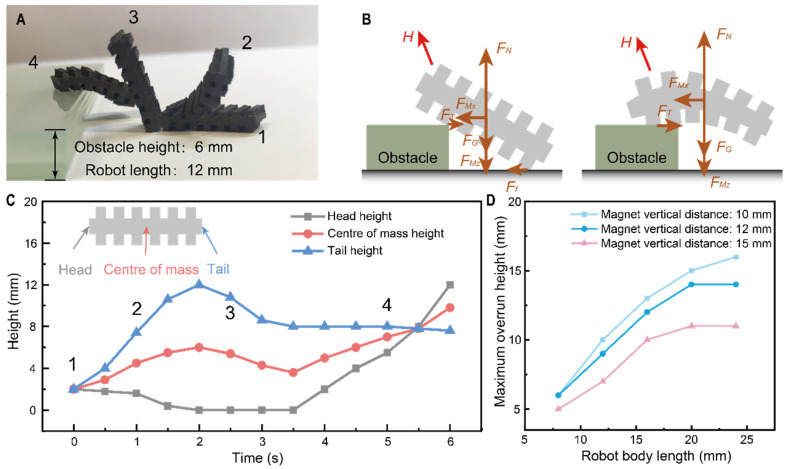
Soft robot overrunning capability based on tumbling motion. (**A**) Soft robot tumbling motion driven by rotating magnetic fields. The steps of the overturning movement are 1, 2, 3 and 4. (**B**) Analysis of forces in two tumbling states. In this figure, H means the internal pulling force, F_Mx_ and F_Mz_ are the magnetic pulling force along x and z direction respectively, F_f_ refers the friction force from ground, F_T_ refers the friction force from the obstacle and F_N_ refers the support. (**C**) Changes in the height of head, tail, and center of gravity during the overturn. (**D**) Relationship between soft robot body length and maximum overturning capacity.

The ability to cross barriers can effectively improve the environmental adaptability of robot movements and expand potential applications in harsh environments. As shown in Figure 3A, for a soft robot with a body length of 12 mm, it is possible to roll over obstacles of up to 6 mm at 90° to the ground. Similar to the forward motion, the climbing process is also achieved under magnetic torque. The difference, however, is that during the raising of the center of gravity, a section placed on the obstacle must provide sufficient friction to resist the soft robot’s own gravity (Figure 3B). As shown in Figure 3C, during climbing, after a flip angle of more than 90° (between state 2 and 3), the robot’s center of gravity gradually drops (from the 2 s to the 3.5 s) and then gradually rises as the flip process progresses. During this process, when the other end of the robot leaves the ground, the robot bends due to gravity and the flexibility of the robot itself. This deformation causes the magnetic moment received by the robot to drop, and as the robot’s body length increases, the direction of the magnetic moment even coincides with the direction of the robot’s descent. This result greatly affects the robot’s overturning capability. At the same time, the relationship between robot length and overturning ability needs to be further verified, as the ability to overturn an obstacle is directly related to the robot’s body length (the longer the robot, the higher the top of the obstacle can be grasped). As shown in Figure 3D, as the robot’s body length increases from 6 mm to 25 mm, the overturning ability first increases linearly (6 mm–16 mm) and then stagnates (20 mm–25 mm) due to the decrease in magnetic moment caused by the natural bending of the robot, as described in the previous section. At the same time, as the magnetic field distance increases, the driving capacity of the magnetic moment decreases accordingly; see Figure S2 for the relationship between the magnetic field strength and distance for the permanent magnets we used. In summary, the overturning capacity of the soft robot is related to the body length and the strength of the driving magnetic field. Still, the excellent overturning capacity (0.5 times the body length) and overturning rate (one tumbling cycle, here 1 s) also demonstrate its ability to perform in complex environments. The outstanding overturning ability (0.5 times body length) and overturning rate also demonstrate excellent performance in complex environments.

### 3.4. Locomotion of Carrying Cargo

Tumbling has distinct advantages in terms of speed of movement and passing ability. However, the robot’s ability to carry could significantly expand its biomedical applications, including, but not limited to, precision drug delivery based on carrying capacity and foreign body capture based on grasping capability. The crawling motion of a snail can be thought of as the soft body’s driving part moving with a load (the snail shell) on its back. The constant contact with the ground during the creeping movement provides a high level of resistance to failure (e.g., sliding or disengagement) compared to the tumbling movement. In addition, the low overall undulation of the robot during creeping motion prevents the load from falling due to excessive changes.

**Figure 4:**
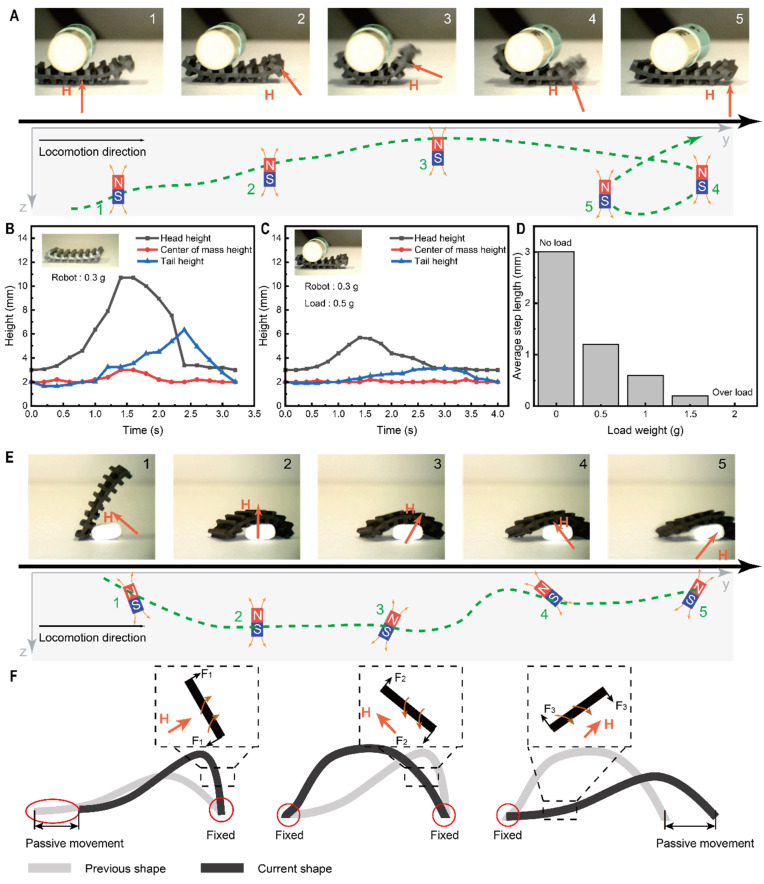
Locomotion analysis of soft robot motion based on imitation of an inchworm and snail. (**A**) Soft robot carrying a capsule that mimics the wriggling of a snail and its corresponding magnetic field direction. The states of motion marked in the optical image (1, 2, 3, 4, 5) correspond to the states of motion of the driven magnet (1, 2, 3, 4, 5). (**B**) The head, tail, and center of gravity of the soft robot vary in height when wriggling without a load. (**C**) The head, tail, and center of gravity of the soft robot vary in height when wriggling with a load. (**D**) Maximum carrying capacity of soft robots. (**E**) Soft robot carrying a pill that mimics the wriggling of an inchworm and its corresponding magnetic field direction. The states of motion marked in the optical image (1, 2, 3, 4, 5) correspond to the states of motion of the driven magnet (1, 2, 3, 4, 5). (**F**) Analysis of the forces in the imitation of a looper movement.

When the applied magnetic field is not strong enough to provide sufficient magnetic torque to drive the soft robot in a fulcrum motion on one side, the side is partially lifted in the direction of the magnetic field, as shown in Figure S5. As shown in Figure 4A, the crawling motion of the soft robot can be controlled by adjusting the position and angle of the permanent external magnet. Initially, the magnet is located directly below the robot, and the robot will adhere to the ground. As the permanent magnet moves forward and upwards, the soft robot lifts one end so that its foot is aligned with the magnetic flux while moving forward under the pull of the magnetic field. The attachment to the ground is then restored as the permanent magnet moves away. The change in the height of the head, tail, and center of gravity during the overall movement is shown in Figure 4B,C. It can be seen that the height change decreases when the capsule is loaded (the maximum center of gravity drops from ~6 mm to ~2.5 mm), avoiding the problem of falling goods during transport. Another important indicator of load carrying capacity is shown in Figure 4D, where the average step length decreases (from 3 mm to 0.2 mm) as the load weight increases (from 0 g to 1.5 g), and the robot no longer has the ability to move when the weight increases further to 2 g.

In addition to mimicking the writhing of a snail, the robot can also mimic the motion of an inchworm to grasp drugs, as shown in Figure 4E. The magnetic field for this movement is generated by a permanent magnet. As shown in Figure 4F, after performing a tumbling motion until it touches the target (Position 1), the soft robot first covers it, forming an arch (Position 2), and then as the permanent magnet moves towards the right foot of the robot. In contrast, the right side of the robot arches while the right endpoint is fixed, causing the left endpoint to move forward (Position 3). This is mainly due to the magnetic moment at this point exerting downward pressure on the right end of the robot, thus increasing the static friction between the contact point and the substrate and fixing its position. For the left-hand part, the magnetic moment generated is small due to the weak magnetic field, and the main movement is due to the pulling force generated by the soft robot deformation. As the permanent magnet moves to the right, the angle between the internal magnetic moment and the horizontal direction is greater than the angle of the external magnetic field, which corresponds to an upward thrust on the right end, reducing the static friction, and causing the right end to advance (Position 4)(Figure S6). Finally, the permanent magnet is moved away, causing the soft robot to return to a neutral position. Based on the above process, the direction of the robot’s crawl can easily be controlled by changing the direction of the permanent magnet’s movement.

Specifically, as the magnet moves from the soft robot’s lower left to the lower right, it approaches the right end of the robot, which exerts a greater attractive force, thus increasing the static friction between the contact point and the ground and fixing its position. For the left part of the soft robot, the magnetic moment generated causes it to rotate counterclockwise, reducing the friction between it and the ground, and in an overall elastic deformation, being dragged into motion (Figure S7). After lifting the applied magnetic field, the robot resumes its initial position as the friction from the ground ensures the fixation of both ends.

### 3.5. Movement of the Soft Robots over a Complex Terrain and their Robustness

**Figure 5:**
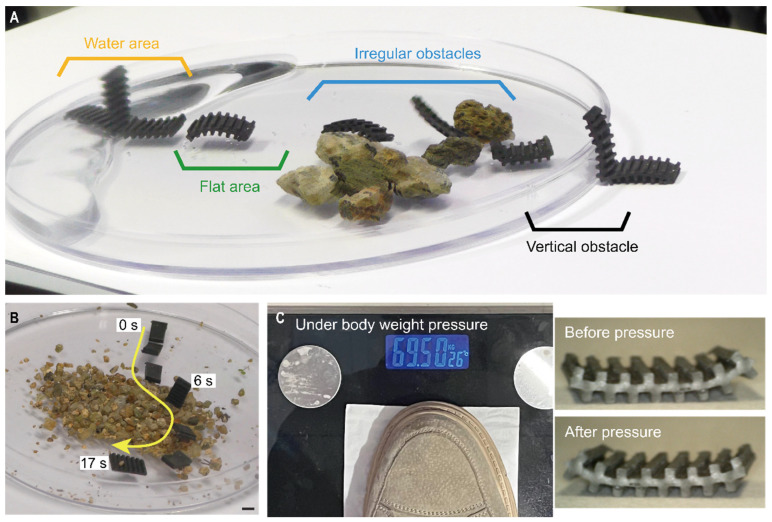
Terrain traversal capability and robustness of the soft robots. (**A**) Soft robot moves on wet ground, flat ground, gravel obstacles, and vertical obstacles, respectively. (**B**) Soft robot steering ability on gravel. (**C**) After adding the weight of an adult man stepping on it, the soft robot recovered as before.

To further validate the soft robot’s adaptability to the environment, we demonstrated movement on wet surfaces, rough surfaces, irregular obstacles, and vertical obstacles, which are relatively harsh environments for a soft robot. As shown in Figure 5A, thanks to the multiple legs, the soft robot can move freely in a variety of complex environments. Time-lagged images show that the soft robot can achieve an effective tumbling motion on wet surfaces, moving 60 mm forward in 6 s. For rough surfaces with sand and stones, the situation is even harsher and more severe. On the one hand, irregular gaps between the sand and stones can easily cause the robot to get caught in them, and on the other hand, the rough shape of these sand or stone surfaces reduces friction. The tumbling motion, on the other hand, allows the robot to use the minimum contact area and ignore changes in height. Under the action of a magnetic field, the milli-inch soft robot can move 60 mm on a gravel surface in 5 s. The final vertical and narrow edge at the edge of the Petri dish, which is 5 mm high, can be easily tumbled over by the soft robot. This series of movements demonstrate the soft robot’s ability to adapt well to the terrain. In addition, we also verified the soft robot’s ability to turn on gravel surfaces, as shown in Figure 5B. The harsh environment also had no effect on the robot’s turning performance.

Robustness is also crucial for the survival of insects. Due to its soft and deformable exoskeleton, a cockroach can withstand loads up to 900 times its weight without injury. Our soft robot also has excellent robustness due to its simple structure and the fact that it is made entirely of soft materials. As shown in Figure 5C, the robot could continue working without significant deformation (Figure S8) after being stepped on by an adult (70 kg), demonstrating good robustness.

## 4. Conclusions

By mimicking several different forms of animal locomotion, we present a fast and highly terrain-adaptive soft robot that can be used for potential application in environmental exploration, structural inspection, drug delivery, and sampling. Despite its simple structure and ease of fabrication, several different forms of locomotion and functions are achieved by adjusting the direction and magnitude of the applied magnetic field.

Despite advances in terrain adaptation and locomotion capabilities, there is still room for progress in designing soft robot structures and internal magnetization orientations. The accuracy of the magnetization can also be further improved, allowing for the development of newer movement patterns and smoother switching between different movement patterns. In conclusion, the insect-scale soft robot with its high robustness opens up a new avenue for constructing bionic robots, and there is still much room for improvement.

## Data Availability

The data that support the plots within this paper and the other findings of this study are available from the corresponding author upon reasonable request.

## Supplementary Materials

The following supporting information can be downloaded at: https://www.mdpi.com/article/10.3390/mi13101578/s1, Figure S1: Optical images of dispersions before and after curing Figure S2: Internal magnetic chain distribution of the soft millirobot; Figure S3: SEM images of the robot’s cross-sectional view; Figure S4: Measured magnetic flux density (black dots) with respect to the distance from the surface of the permanent magnet along its axis. The curve is the numerical simulation based on the magnetic field model by MATLAB; Figure S5: Lifting angles of flexible robots under different magnetic fields. The lifting angle of the legged structure is greater than that of the legless structure at the same magnetic field; Figure S6: Analysis of friction during crawling; Figure S7: Analysis of friction during creeping; Figure S8: Cycling compression test of the soft robot (Load: 70 kg); Video S1: Multiple locomotion modes for soft millirobot.
