# Supplementary material for: An Insect-Inspired Terrains-Adaptive Soft Millirobot with Multimodal Locomotion and Transportation Capability
Source: Micromachines (Basel). 2022 Sep 22;13(10):1578. doi: 10.3390/mi13101578 (PMC9610805; doi:10.3390/mi13101578)
Supplement: Supplementary file 1 [file micromachines-13-01578-s001.zip › micromachines-1856468-supplementary.pdf]

Supplementary Materials for

An insect-inspired terrains-adaptive soft millirobot with multimodal locomotion and transportation capability

Han Huang[1], Yu Feng[1], Xiong Yang[1], Liu Yang [1], Yajing Shen[1, 2, *]

Correspondence to: yajishen@cityu.edu.hk

This PDF file includes:

Supplementary Text
Figures. S1 to S8
Video. S1

Supplementary Text

Analysis of the curing process

In nanoparticle suspensions, powder agglomeration is an inherent feature of fractions. When agglomeration develops to a certain extent the system will settle and delaminate and fail. The settling force F_1 on particles in suspension can be expressed as:

$$F_1 = \frac{1}{6}\pi d^3(\rho - \rho_0)g$$

Where: ρ_0 is the base fluid density; d is the particle equivalent diameter; ρ is the particle density; g is the medium gravitational acceleration. According to stokes' law, the particle is subjected to a settling resistance F_2 of：

$$F_2 = 3\pi\epsilon d v_0$$

Where: ϵ is the dynamic viscosity of the base fluid; v_0 is the settling velocity. When the particles are steadily suspended or falling at a uniform rate then we have: $F_1 = F_2$ which gives:

$$v_0 = \frac{d^2(\rho - \rho_0)}{18\epsilon g}$$

As can be seen from the above equation, the settling velocity of particles in suspension is proportional to the square of their equivalent diameter; the larger the particles, the faster they settle. The greater the difference between the base liquid and particle densitay of the suspension, the faster it settles. The higher the viscosity of the base liquid of the suspension, the slower it settles. In the case of Ecoflex suspensions with magnetic particles, settling can be slowed down by using a 5000 mesh powder and a high viscosity of Ecoflex. At the same time, the viscosity of Ecoflex increases during curing until it becomes an elastic solid, effectively avoiding settling problems.

To further verify the homogeneity of the material, SEM images were taken of the robot's profile, as shown in Fig. S4, with overall homogeneity and no settling. Further low concentration comparison experiments are shown in Fig. S5. After 40 minutes of curing, no colour change or delamination occurred, and it can be assumed that no significant settling problems occurred during the curing process.

Friction analysis during motion

As described in the manuscript, the motion of a magnetic soft robot is achieved by a combination of magnetic pull and torque forces. The part of the robot in contact with the ground is subject to both traction from the magnetic field and friction from the ground. The action of the magnetic pull causes deformation of the legs of the flexible robot. When the magnetic traction force is greater than the friction force, the flexible robot can move forward with a certain acceleration. As the robot moves along the x-axis, the dynamic model can be written as:

$$F_f = \mu(mg + F_{mZ} - F_T sin\alpha + mz)$$

Supplementary Figures

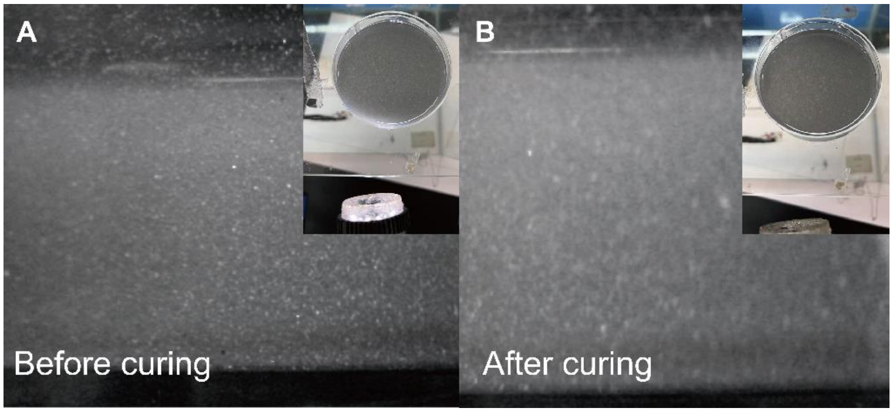

Figure S1. Optical images of dispersions before and after curing

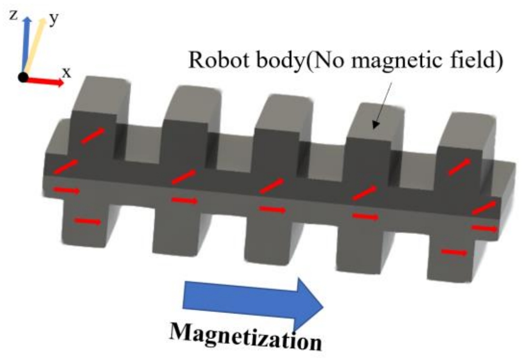

Figure S2. Internal magnetic chain distribution of the soft millirobot

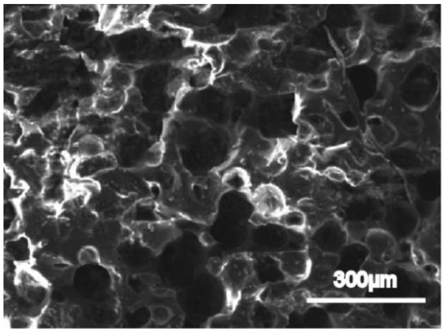

Figure S3. SEM images of the robot's cross-sectional view

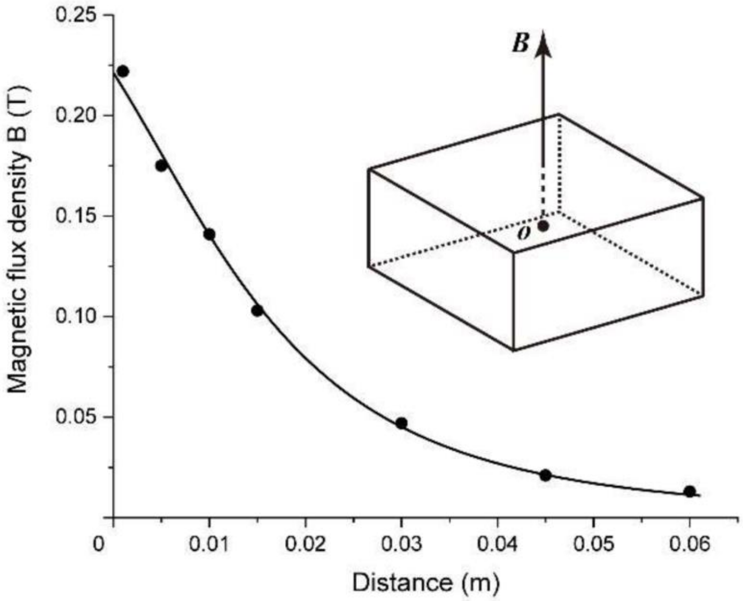

Figure S4. Measured magnetic flux density (black dots) with respect to the distance from the surface of the permanent magnet along its axis. The curve is the numerical simulation based on the magnetic field model by MATLAB.

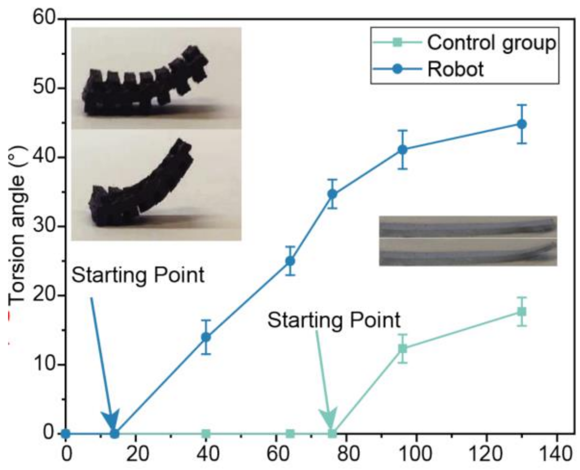

Figure S5. Lifting angles of flexible robots under different magnetic fields. The lifting angle of the legged structure is greater than that of the legless structure at the same magnetic field.

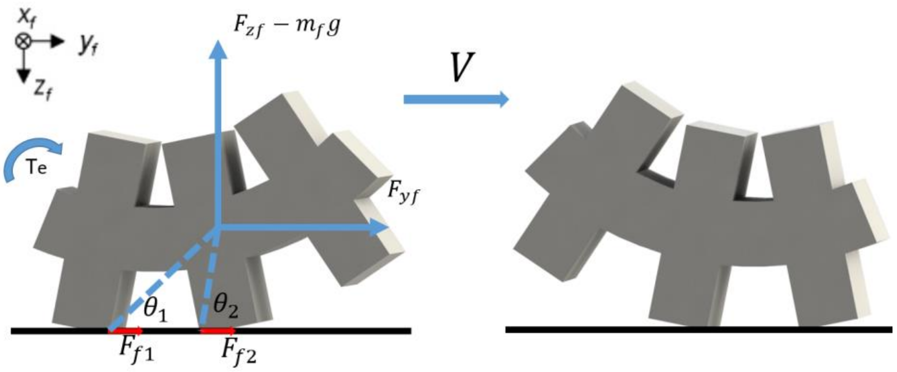

Figure S6. Analysis of friction during crawling

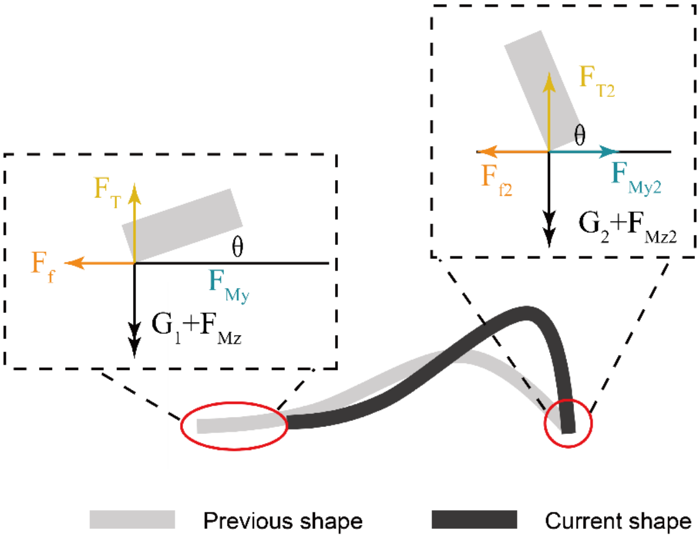

Figure S7. Analysis of friction during creeping

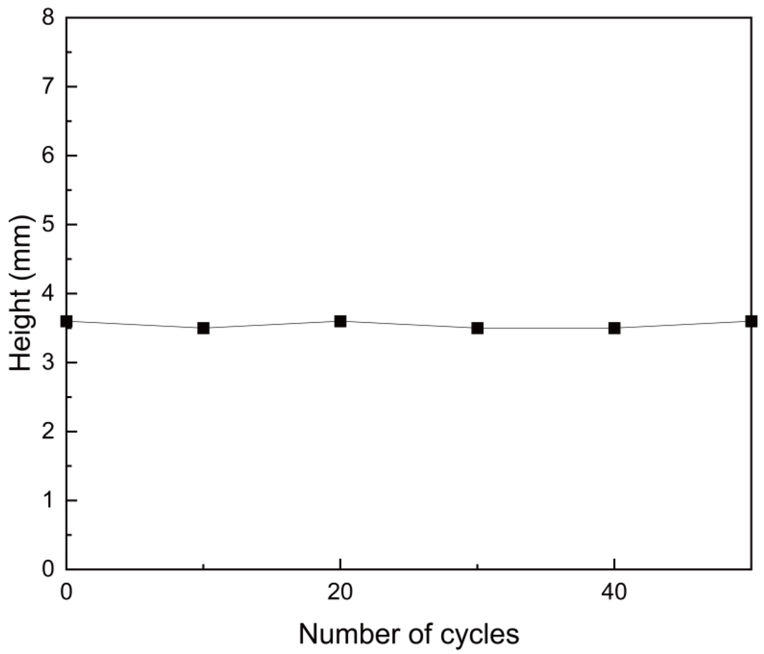

Figure S8. Cycling compression test of the soft robot (Load: 70kg)
